# Optimising physiotherapy for people with lateral elbow tendinopathy – Results of a mixed-methods pilot and feasibility randomised controlled trial (OPTimisE)

**DOI:** 10.1016/j.msksp.2023.102905

**Published:** 2024-02

**Authors:** M. Bateman, A. Skeggs, E. Whitby, V. Fletcher-Barrett, G. Stephens, M. Dawes, D. Davis, J. Beckhelling, K. Cooper, B. Saunders, C. Littlewood, B. Vicenzino, N.E. Foster, J.C. Hill

**Affiliations:** a University Hospitals of Derby & Burton NHS Foundation Trust, Derby, UK; b School of Medicine, Keele University, UK; c Derby Clinical Trials Support Unit, Derby, UK; d Sheffield Teaching Hospitals NHS Foundation Trust, Sheffield, UK; e Royal Orthopaedic Hospital, Birmingham, UK; f Patient Representative, Derby, UK; g Edge Hill University, UK; h University of Queensland, Brisbane, Australia; i STARS Education and Research Alliance, Surgical Treatment and Rehabilitation Service (STARS), The University of Queensland and Metro North Health, Brisbane, Australia

## Abstract

**Background:**

The OPTimisE intervention was developed to address uncertainty regarding the most effective physiotherapy treatment strategy for people with Lateral Elbow Tendinopathy (LET).

**Objectives:**

To assess the feasibility of conducting a fully-powered randomised controlled trial (RCT) evaluating whether the OPTimisE intervention is superior to usual physiotherapy treatment for adults with LET.

**Design:**

A mixed-methods multi-centred, parallel pilot and feasibility RCT, conducted in three outpatient physiotherapy departments in the UK.

**Method:**

Patients were independently randomised 1:1 in mixed blocks, stratified by site, to the OPTimisE intervention or usual care. Outcomes were assessed using pre-defined feasibility progression criteria.

**Results:**

50 patients were randomised (22 Female, 28 Male), mean age 48 years (range 27–75). Consent rate was 71% (50/70), fidelity to intervention 89% (16/18), attendance rate in the OPTimisE group 82% (55/67) vs 85% (56/66) in usual care, outcome measure completion 81% (39/48) at six-month follow-up. There were no related adverse events. Patients and physiotherapists reported that the OPTimisE intervention was acceptable but suggested improvements to the trial design. 49 patients were recruited from physiotherapy referrals vs one from primary care records. Outcome measure return rates were higher when completed online (74%) compared to postal questionnaire (50%). Exploratory analysis showed improvements in both groups over time.

**Conclusions:**

It is methodologically feasible to conduct a fully powered RCT comparing the clinical and cost-effectiveness of the OPTimisE intervention versus usual physiotherapy treatment. Considering the similar improvements observed in both groups, careful consideration is needed regarding the priority research question to be addressed in future research.

## Highlights

• The OPTimisE lateral elbow tendinopathy intervention was acceptable to patients.
• The OPTimisE intervention was delivered as intended by physiotherapists.
• Recruitment and retention rates within the OPTimisE trial were high.
• A future main trial is unlikely to be a priority given the clinical outcomes.

## 1 Introduction

Lateral elbow tendinopathy (LET), also known as Tennis Elbow, is a common cause of elbow pain affecting sleep and basic daily activities, as well as sports, work and hobbies (Bateman et al., 2023; Sanders et al., 2015; Keijsers et al., 2019). Work absence due to LET was estimated to cost the UK economy £27million based upon data from 2012 (Hopkins et al., 2016). For many, symptoms may resolve over time but 10% of control group participants in clinical trials failed to achieve much improvement or full resolution after one year (Ikonen et al., 2022). Physiotherapy is recommended if symptoms persist after six weeks. (National Institute for Health and Care Excellence) Evidence from two high-quality trials suggests that whilst multi-modal physiotherapy does not influence the long-term outcome for people with LET, it results in faster recovery in the short term (Coombes et al., 2013; Bisset et al., 2006). These studies were conducted between 2002 and 2010, with patients receiving eight treatments over a six to eight week period. Such treatment intensity is challenging to deliver in publicly-funded healthcare systems and considered to be over-burdensome by some patients (Bateman et al., 2021). Wide variations in current physiotherapy treatment provision have been identified that do not align with research evidence (Bateman et al., 2018, Bateman et al., 2019, Bateman et al., 2023). Treatments including ultrasound therapy, massage, acupuncture and shockwave therapy are still used in clinical practice without scientific justification (Tang et al., 2015; Long et al., 2015; Buchbinder et al., 2006; Bisset and Vicenzino, 2015). There is therefore a need to streamline physiotherapy provision, ensuring that treatment protocols are efficient, evidence-based and practical to use.

The OPTimisE intervention was designed in consultation with patients and clinicians to reflect the current evidence base in a way that could be implemented into real-world clinical practice. We combined research evidence with the opinions of patients, physiotherapists with a special interest in LET and physiotherapy service managers, to form a consensus on what the intervention should comprise (Bateman et al., 2021). The OPTimisE intervention has been previously described in full and consists of three elements: condition-specific and general health advice that addresses modifiable risk factors, supported by high-quality printed and online resources; an exercise regimen that empowers the patient to progress or regress their rehabilitation based upon limits of pain deemed acceptable by individual patients; and the provision of a counter-force orthosis (Bateman et al., 2021). Prior to implementation, the OPTimisE intervention needed to be tested in clinical practice to establish firstly, whether it was deliverable and secondly, whether it was effective. We therefore aimed in this study to test the feasibility of conducting a future, fully-powered randomised controlled trial that would evaluate the clinical and cost-effectiveness of the OPTimisE intervention versus usual physiotherapy treatment.

## 2 Method

### 2.1 Trial design

We conducted a parallel two-arm, multi-centre pilot and feasibility randomised controlled trial across three sites. The detailed protocol has previously been published (Bateman et al., 2022a). Recruitment took place between September 2021 and August 2022. The findings are reported following the CONSORT Pilot Trial Checklist (Eldridge et al., 2016).

### 2.2 Feasibility outcomes

Our primary aim was to determine feasibility (criteria shown in Table 1) with reference to the following objectives:

• Consent rate (number consented from those eligible after screening for inclusion/exclusion criteria)
• Intervention fidelity in the intervention group (measured as a binary outcome if participants were given the orthosis, taught the progressive exercise regimen and received advice/education on a minimum of 6 of the 12 specified topics)
• Attendance rate in the intervention group (number of physiotherapy appointments attended from the total appointments booked)
• Outcome measure completion rate at six months post-randomisation

**Table 1:**
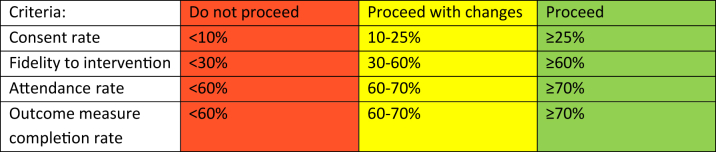
Feasibility criteria for a future main trial.

Recruitment feasibility of 25% was selected based upon 50 patients being recruited from 200 patients referred per year – data that the three sites had provided from historic referral patterns. The fidelity criterion was determined by the research team as no precedent has been set. Attendance rate of 70% was set based upon previously published data for physiotherapy outpatient attendance (Smith and Bateman, 2014). Outcome measure completion rate of 70% was based upon the TATE trial, a UK physiotherapy trial for LET, that had 69% data returns (Chesterton et al., 2013).

The secondary aims were to assess:

• Outcome measure completion rate at 6 weeks and 12 weeks post-randomisation
• Completion of a grip-strength physical measure at two time points using the Squegg device
• Patient-reported outcome measures (PROMs) at baseline, 6 weeks, 12 weeks and 6 months post-randomisation (analysed descriptively)
• Responsiveness to change analysis of individual PROMs questionnaires compared with patient perceived overall treatment effect, to determine the most appropriate PROMs for a future trial
• Adherence to exercise therapy treatment (measured using a self-reported exercise diary and Exercise Adherence Rating Scale (EARS))(Newman-Beinart et al., 2017)
• Acceptability of the optimised physiotherapy treatment package and trial processes, investigated through the nested qualitative study

### 2.3 Participants

We piloted two methods of participant identification: screening of General Practice (GP) computer records and screening of referrals at physiotherapy sites. Those patients identified from GP records were sent a self-screening checklist and invitation letter to contact the trial team if they wished to be considered. Their GP was then asked to refer them to their local physiotherapy site. All physiotherapy referrals for elbow pain were screened at one of three National Health Service (NHS) physiotherapy sites, in either Birmingham, Derby or Sheffield. Patients deemed potentially eligible were sent a patient information sheet (PIS) then telephoned by the site principal investigator (PI) to check eligibility and invite for clinical assessment screening. Inclusion criteria: adults aged 18 or over; physiotherapist-diagnosed tennis elbow which included pain on palpation of the common extensor origin and on gripping; either a positive Cozen's, Mills', or Maudsley's test (Zwerus et al., 2018). Exclusion criteria: a recent history of significant trauma to the affected limb, e.g. a fall on an outstretched hand; previous diagnosis of inflammatory arthritis or gout; previous diagnosis of osteoarthritis of the affected elbow; neurological symptoms in the affected limb correlating with onset of elbow pain, e.g. loss of sensation in the hand; co-existing neck pain and stiffness that started at a similar time to the elbow symptoms; inability to understand English or lacking capacity for informed consent; currently enrolled in another health-related research trial.

### 2.4 Randomisation

Following assessment, we invited eligible patients to provide informed consent, complete baseline questionnaires and they were then randomised via an online service (Sealed Envelope™) using 1:1 allocation in mixed blocks or 2 and 4, stratified by treatment site. We also asked if they consented to be contacted about participation in the qualitative study nested within the trial, following their course of treatment.

### 2.5 Interventions

We allocated participants to receive either the OPTimisE intervention or usual physiotherapy care. We did not standardise usual care in this pragmatic trial but we recorded treatments received by participants, to allow comparison with the OPTimisE intervention. Physiotherapists providing usual care treatment had no restrictions on the treatments they could offer. Physiotherapists provided treatments for one intervention arm only, to minimise contamination. Those providing usual care did not receive training in the OPTimisE intervention.

The OPTimisE intervention consisted of three elements: condition-specific and general health advice, supported by printed and online resources; a progressive exercise regime working within limits of pain deemed acceptable by individual patients; and the provision of a counter-force orthosis. Exercises were progressed when the existing exercise was no longer painful, or regressed if the exercise provoked unacceptable levels of pain. The OPTimisE Intervention Handbook is supplied as a supplementary file, providing full details of the intervention.

At the first appointment, those in the OPTimisE arm were provided with a Patient Manual containing advice/education material, exercise instructions and a password to access online resources. Advice and education topics were then discussed with the physiotherapist. Participants were supplied with an orthosis (Epi-Hit® Classic), as a means of providing short-term symptomatic relief, and instructed how to fit it correctly, then taught an exercise regimen that they could progress or regress based upon their symptom response. Follow-up appointments, to review progress, discuss advice/education topics further and review/adjust exercises, were arranged at the discretion of the physiotherapist but guidance was that appointments should be spaced at least four weeks apart, as recommended by patients during the intervention design. Appointments could be face-to-face, online or by telephone.

### 2.6 Blinding

Due to the nature of the interventions, neither participants nor physiotherapists could be blinded. Data analysis was not masked to allocation.

### 2.7 Data collection

We gathered patient-reported data using a questionnaire containing the recommended Core Outcome Set for LET (Patient-Rated Tennis Elbow Evaluation (PRTEE),^1^ time off work (measured in days), pain-free grip-strength, Numerical Rating Scale (NRS)^2^ measuring pain on gripping, (Bateman et al., 2022b) plus the Global Perceived Effect scale (GPE-11),^3^ (Kamper et al., 2010), Tampa Scale of Kinesiophobia (TSK-11),^4^ (Bisson et al., 2022), Patient Self-Efficacy Questionnaire (PSEQ),^5^ (Nicholas, 2007), EuroQol 5D5L,^6^ (Herdman et al., 2011) maximum grip strength and EARS questionnaire.^7^ (Newman-Beinart et al., 2017). For the grip-strength measurements, we piloted the use of an electronic grip-measuring device (Squegg™, https://mysquegg.com) that connects to an application on the participant's smartphone or tablet. The Squegg is a US Food and Drug Administration approved dynamometer. We gave participants in the OPTimisE group a Squegg after randomisation, to capture grip-strength data at all time points. However, to ensure that usual care participants did not use it as part of their treatment, they were only sent the Squegg by post in advance of their final six-month follow-up questionnaire. Grip measurements were taken in neutral forearm rotation, with the elbow at 90°. The mean value was taken from three measurements.

Participants were given the choice of receiving questionnaires by post or online, using the Amplitude Pro-One™ system (https://amplitude-clinical.com/) and were sent questionnaires at 6-weeks, 12-weeks and 6-months post-randomisation. We telephoned or sent reminders by email to participants using the postal service if they failed to return their questionnaires after two weeks. The Amplitude system sent automated email and SMS text reminders to users of the online system after one and two weeks if data was not returned. We amended the protocol mid-way through the trial to allow the site PI to telephone participants who had not returned questionnaires, to collect minimum data about adverse events and the primary outcome (PRTEE) responses. We implemented this at 6-month follow-up, due to low data returns at 6-weeks and 12-weeks. We asked participants in the OPTimisE intervention group to complete a daily exercise diary to collect data about exercise adherence.

## 3 Analytical methods

### 3.1 Quantitative data analysis

Descriptive statistics were used to summarise the distribution of baseline variables across each of the randomisation groups. The continuous baseline variables (e.g. age) were reported with means and 95% confidence intervals (95% CI), if shown to be normally distributed, otherwise were reported with medians and Interquartile Ranges (IQR). The categorical variables (e.g. sex) were reported with frequencies & percentages. Similarly, we analysed data descriptively to explore the outcome measure scores in the intervention and control groups at baseline and follow-up, to explore changes in LET health status over time. The study was not powered for analysis of results between groups. We also assessed external responsiveness to change of PROMs using Spearman's rank correlation, with GPE-11 scores as the anchor. SPSS Statistics software (version 27) was used for the analysis.

### 3.2 Qualitative interviews

At least three months after randomisation, we sent a purposive sample of patients a PIS and invitation letter to take part in a qualitative interview. We followed this up by telephone or email to gain provisional consent and organise a mutually convenient time. Similarly, physiotherapists involved in the trial were approached at the end of recruitment. Interviews were conducted face-to-face, by telephone or video-conference via Microsoft Teams, at the participant's preference. We audio-recorded all interviews and asked participants to formally consent verbally after reading a consent form. Interview recordings were transcribed verbatim using an online service (https://www.universitytranscriptions.co.uk/). All transcriptions were checked for accuracy and any uncertainties were resolved by relistening to the original recording. Anonymised interview transcripts were analysed using inductive thematic analysis (Braun and Clarke, 2006). Transcript coding was performed using NVivo 12 software. Interviews continued until data saturation was reached, assessed in terms of ‘informational redundancy’, the point at which additional data no longer offers new insights (Sandelowski and Given, 2008). Data from the qualitative study were used to assess acceptability from the perspectives of both patients receiving the OPTimisE intervention and physiotherapists delivering it (Bateman et al., 2023). Feedback related to trial processes was used to refine the method of a future fully-powered trial.

### 3.3 Data management

Data were collected using a mix of paper and electronic methods. Where possible a patient ID number was used rather than identifiable information. Data from paper forms were transcribed into an electronic database in Microsoft Excel stored on secure NHS servers. Paper hard copies were stored at Derby CTSU and in the relevant Investigator Site Files (ISF). Study documentation was stored securely to maintain participant confidentiality and study data integrity.

Electronic data captured at trial sites was uploaded to a secure electronic ISF on Microsoft Sharepoint. Online outcome data collection was managed by Amplitude Clinical in ISO27001 Tier 3+ data centres approved for use by the NHS.

## 4 Results

We recruited the target of 50 patients (stipulated by the funder) within the allocated 12-month time-period. Baseline data are displayed in Table 2.

**Table 2:** Summary of baseline data.

SUMMARY OF BASELINE DATA		OPTimisE Intervention (n = 24)	Usual Care Treatment (n = 26)
**Age** mean (SD)		51 (9.4)	46 (8.4)
**Body Mass Index** median (interquartile range values)		26.30 (24.47–30.72)	26.43 (23.49–29.16)
**Duration of symptoms months** median (interquartile range values)		7 (4-12)	7 (4.75–12)
**Sex** n (%)	Male	12 (50)	16 (62)
	Female	12 (50)	10 (38)
	Other	-	-
	Preferred not to say	-	-
**Ethnicity** n (%)	White British	21 (88)	19 (73)
	White Other	-	2 (8)
	Mixed	-	1 (4)
	Indian	1 (4)	2 (8)
	Pakistani	-	1 (4)
	Sri Lankan	1 (4)	-
	Filipino	-	1 (4)
	Kosovar	1 (4)	-
**Hand Dominance** n (%)	Right	23 (96)	24 (92)
	Left	1 (4)	2 (8)
**Affected Side** n (%)	Right	17 (71)	17 (65)
	Left	6 (25)	9 (35)
	Bilateral	1 (4)	-
**Smoking Status** n (%)	Smoker	4 (17)	4 (15)
	Non-smoker	11 (46)	10 (39)
	Ex-smoker	9 (38)	11 (42)
	Occasional smoker	-	1 (4)
**Questionnaire Delivery Preference** n (%)	Paper	6 (25)	5 (19)
	Online	18 (75)	21 (81)

**Fig. 1:**
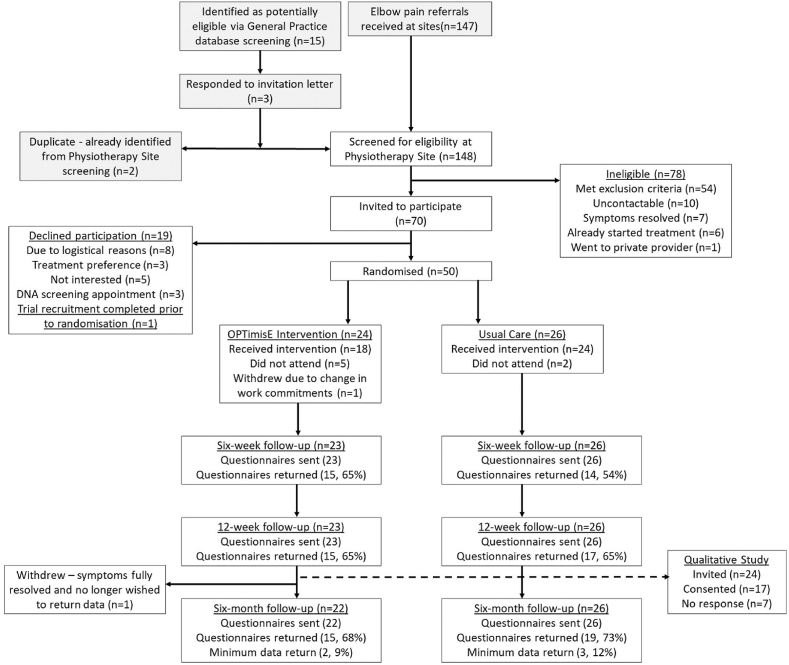
CONSORT diagram.

The CONSORT diagram is shown in Fig. 1.

### 4.1 Feasibility outcomes

**Fig. 2:**
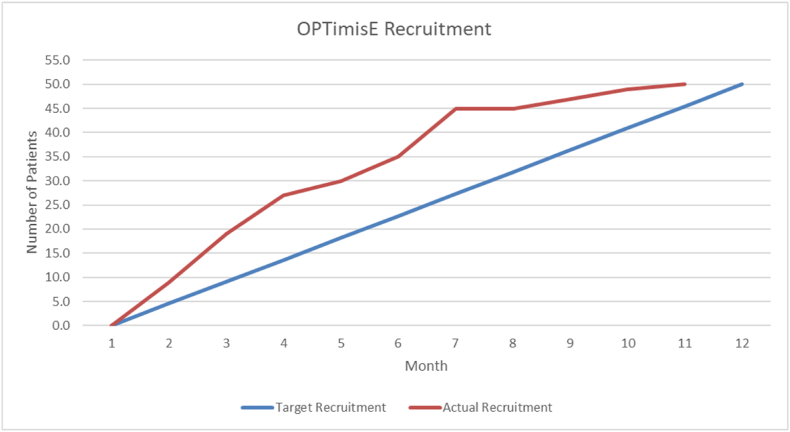
Recruitment graph.

We enrolled the target of 50 participants six weeks ahead of schedule (as shown in Fig. 2), from a pool of 70 identified eligible participants, giving a consent rate of 71%. Two participants in the OPTimisE group subsequently withdrew: one prior to commencing treatment, due to moving away from the area because of a change of work; another after returning their 12-week questionnaire, stating that their symptoms had fully resolved but they did not wish to return the final questionnaire. All participants in the OPTimisE group that attended received the intervention in full, except for two, who received the orthosis and progressive exercise but only five advice/education topics instead of six, resulting in intervention fidelity of 89% (16/18). The attendance rate at all planned sessions in the OPTimisE intervention group was 82% (55 attendances from 67 booked appointments), compared with 85% (56 attendances from 66 booked appointments) in the usual care group. Patients typically waited between 2 and 8 weeks from consent to receive their first treatment. Patients in the OPTimisE group attended a mean of 3.1 sessions, compared to 2.3 sessions in the usual care group. Five participants in the OPTimisE intervention group failed to attend their first treatment session, so did not receive the intervention. Two participants in the usual care group failed to attend their first treatment session, so did not receive the intervention. Outcome measure completion, using the PRTEE as the minimum data collection tool, was 81% (39/48) at six-month follow-up. All four feasibility criteria met the threshold to proceed to a main trial.

### 4.2 Secondary outcomes

Of the two patient recruitment methods, physiotherapy referral screening provided 49 participants, whereas only 1 participant was recruited from GP record screening (having identified 15 potentially eligible people).

The outcome measure return rate at six weeks was 59% (66% online vs 36% paper); at 12 weeks was 65% (68% online vs 55% paper); at six months was 81% (28/38 online versus 5/10 paper, plus four minimum data telephone collections and two paper returns after requests from participants who had originally opted for online). 27/39 (69%) of 6-month data returns included grip-strength measurements using the Squegg device.

The descriptive analysis of the patient-reported outcome measures is presented in Table 3.

**Table 3:** Descriptive analysis of patient-reported outcome measures.

Secondary ANALYSIS Median (interquartile range values) Sample size n	Baseline		6 weeks		12 weeks		6 months	
	OPTimisE Group	Usual Care Group	OPTimisE Group	Usual Care Group	OPTimisE Group	Usual Care Group	OPTimisE Group	Usual Care Group
PRTEE	*46.25 (40.5-69.625) n=24*	*45 (36.5-62.125) n=26*	*42.5 (24-71) n=15*	*40 (17*.*75-55.5) n=13*	*30 (10.5-53.5) n=15*	*20.5 (10.25-59.75) n=17*	*12.5 (5.5-37.25) n=17*	*8.5 (3-27.625) n=22*
% Achieving MCID on PRTEE	*-*	*-*	*-*	*-*	*7/15 (47%)*	*9/17 (53%)*	*12/17 (71%)*	*20/22 (91%)*
Pain on gripping (NRS)	*6.50 (4.25-8) n=24*	*7 (4-7.25) n=26*	*5 (4-8) n=15*	*5 (2.75-6.5) n=14*	*4 (1-6) n=15*	*4 (1.5-6) n=17*	*2 (1-3.75) n=16*	*2 (1-3.5) n=21*
GPE-11	*-*	*-*	*1 (0-2) n=15*	*0 (-0*.*5-2.5) n=13*	*2 (1-4) n=15*	*1 (0-4) n=17*	*3 (1.25-5) n=16*	*4 (1-5) n=20*
% Scoring +4 or +5 on GPE-11	*-*	*-*	*-*	*-*	*4/15 (27%)*	*6/17 (35%)*	*7/16 (44%)*	*12/20 (60%)*
TSK-11	*25.5 (19.5-28.75) n=24*	*25.5 (22-31) n=26*	*23 (19-27) n=15*	*25 (20-28.5) n=13*	*19 (17-25) n=15*	*24 (19-27) n=17*	*19 (14-25.25) n=14*	*20 (16-26) n=19*
PSEQ	*41.5 (37-53) n=24*	*38.5 (31.25-48.25) n=26*	*51 (36-59) n=15*	*45 (41-55.5) n=13*	*52 (48-59) n=15*	*47 (30-59.5) n=17*	*56 (47.5-60) n=13*	*56 (37-60) n=19*
EQ5D5L index	*.800 (.570-.864) n=24*	*.806 (.717-.866) n=26*	*.768 (.624-.837) n=15*	*.768 (.579-.816) n=13*	*.795 (.736-.837) n=15*	*.706 (.535-.816) n=17*	*.795 (.704-.888) n=13*	*.837 (.704-1.000) n=19*
EQ5D5L Health status	*80 (71.25-90) n=24*	*77.5 (63.75-81.25) n=26*	*84 (79-90) n=15*	*75 (62-81) n=13*	*89 (70-94) n=15*	*78 (69-89.5) n=17*	*89 (74.5-91.5) n=13*	*89 (70-90) n=19*
EARS	*-*	*-*	*15.5 (12-22.5) n=14*	*19 (16-24) n=13*	*15.5 (9.5-21.5) n=14*	*16 (12-20.75) n=16*	*13 (8-21) n=13*	*16 (12-23) n=19*
Pain free grip-strength (lbs)	*25 (15-39) n=24*	*27 (20-48) n=26*	*39 (34-58) n=13*	*-*	*44 (34-55) n=13*	*-*	*44 (32-58) n=11*	*44 (27-54) n=16*
Maximum grip-strength (lbs)	*48 (36-58) n=24*	*50 (40-59) n=26*	*52 (43-64) n=12*	*-*	*52 (47-62) n=13*	*-*	*52 (40-65) n=11*	*52 (43-57) n=16*
Time off work (days)	*0 (0-0) n=24*	*0 (0-0) n=26*	*0 (0-0) n=14*	*0 (0-0) n=13*	*0 (0-0) n=12*	*0 (0-0) n=16*	*0 (0-0) n=15*	*0 (0-0) n=19*

The external responsiveness of individual outcome measures, correlated against the GPE-11 anchor, is displayed in Table 4. The PRTEE and NRS for pain on gripping demonstrated the highest correlation with perceived treatment effect at both 12-week and six-month follow-up. Only four participants reported taking time off work at baseline and only one at six-month follow-up, so this domain was not analysed due to lack of data.

**Table 4:** External responsiveness of outcome measures to GPE-11 anchor.

	12 weeks	6 months
PRTEE	−0.800 (p < 0.001)	−0.839 (p < 0.001)
NRS: Pain on gripping	−0.781 (p < 0.001)	−0.852 (p < 0.001)
TSK-11	−0.516 (p = 0.002)	−0.540 (p = 0.001)
PSEQ	0.673 (p < 0.001)	0.714 (p < 0.001)
EQ5D5L (index)	0.583 (p < 0.001)	0.691 (p < 0.001)
EQ5D5L (health status)	0.544 (p = 0.001)	0.366 (p = 0.040)
Pain Free Grip-strength		0.499 (p = 0.008)
Maximum Grip-strength		0.410 (p = 0.034)

Exercise adherence score (median (IQR values)), measured using the EARS questionnaire, at 12 weeks was 15.5 (9.5–21.5) in the OPTimisE group compared to 16 (12–20.75) in the usual care group; at six months 13 (8-21) and 16 (12-23) respectively. Only 6/18 (33%) participants who received the OPTimisE intervention returned their exercise diaries, reporting median adherence of 81% (IQR 74-93).

The review of clinical report forms from the usual care group showed that all patients received basic advice about LET and were taught exercises. Few were provided with advice related to lifestyle factors or modifiable risk factors. Exercises often lacked a clear dosing strategy or progression. Three patients received manual therapy treatment from the physiotherapist and one was taught to perform self-administered manual therapy. No patients were provided with an orthosis, although two patients requested advice on how to fit orthoses that they had previously purchased themselves.

No related adverse events were reported. One participant was involved in a road traffic collision during their period of treatment. They did not sustain serious injuries and were able to continue their trial participation.

From the qualitative interviews, patient and physiotherapist participants both found the OPTimisE intervention to be acceptable and physiotherapists perceived key differences to usual care, related to exercise selection, dose, educational content and provision of the orthosis. The slowing of recruitment after month seven was perceived to be due to a change in referral patterns, with more patients with LET managed by First Contact Practitioners (FCPs)^$^, rather than being referred to outpatient physiotherapy clinics. We identified improvements that could be made to the trial processes to reduce administrative burden, increase support for physiotherapists, improve return rate of outcome questionnaires, and provide language translation to increase accessibility for under-served communities. Details of these findings can be found in a separate publication (Bateman et al., 2023).

[^$^Footnote: FCPs are primary care healthcare professionals, typically physiotherapists in the context of musculoskeletal conditions, who assess and manage patients instead of a General Practitioner.]

## 5 Discussion

Our results suggest that it is feasible to conduct a full-scale trial to compare the clinical and cost-effectiveness of the OPTimisE intervention compared with usual NHS physiotherapy care. We successfully recruited to target ahead of schedule, but the number of eligible patients identified was lower than predicted based upon site referral data at the planning stage of the project. This was offset by consent rates being far greater (71%) than the conservative feasibility target (25%) set *a priori*. The low eligibility numbers may have been in part due to the COVID-19 pandemic and also due to the rollout of FCP services across the English healthcare system. The qualitative interview findings suggest that more patients with LET are being managed in community settings by FCPs rather than being referred to outpatient physiotherapy clinics. The attendance rate at all planned sessions in the OPTimisE intervention group was 82% (55/67 sessions), with five participants not receiving the intervention. This compared to 85% (56/66 sessions) in the usual care group, with two participants not receiving any treatment. The patients interviewed in the qualitative study had positive views with regard to randomisation and did not express strong preferences for their treatment group allocation, however a potential source of bias within the qualitative study is that none of the patients who failed to attend for treatment agreed to be interviewed, despite being approached. It is possible that those who did not attend were dissatisfied with their group allocation resulting in their non-attendance. Alternatively, the wait of 2–8 weeks from consent to receiving their first treatment might have been a factor.

In terms of fidelity, the OPTimisE intervention was delivered as intended to the majority of patients (89%). The pre-defined quantitative criteria for fidelity were binary (i.e. fidelity was achieved or not) but in two cases, fidelity was not achieved because physiotherapists only delivered five of the twelve advice/education topics instead of the six required to satisfy the fidelity criteria. The other remaining criteria, related to exercise prescription and provision of the counterforce brace, were all satisfied.

Of the two patient identification methods, the screening of referrals at physiotherapy clinics was most successful, accounting for 49/50 patients recruited. The database screening at GP practices only generated three expressions of interest, with two of those already identified from referral screening, so this method is unlikely to be worthwhile within a future main trial.

We found that questionnaire returns were low at six-week follow-up (59%). The follow-up questionnaires were sent at time points determined from the date of randomisation. Some patient participants told us in the interviews that they had not returned the questionnaire due to the fact that they had not yet started treatment or only recently started treatment at the time the questionnaire was sent, due to waiting times for initial physiotherapy appointments. Returns increased by 12-week follow-up to 65% but did not meet the feasibility threshold. We introduced a protocol amendment to allow telephone reminders and minimum data collection at this stage which, coupled with the pre-agreed £20 voucher incentive for six-month data return, resulted in an 81% response, surpassing the feasibility threshold.

When we examined the responsiveness of the different PROMs included, the PRTEE measure of function and NRS for pain on gripping showed the highest correlation with patient perceived overall treatment effect. The PRTEE is the recommended primary outcome measure in the core outcome set for LET and the NRS for pain on gripping is recommended for interim use, pending psychometric evaluation (Bateman et al., 2022b). Our findings suggest that it has similar external responsiveness to the PRTEE. During the qualitative interviews, patient participants were receptive to the idea of adding a monthly SMS text message asking them to report their level of pain on gripping. They felt that this might act as a reminder that they were still part of the trial and provide additional data regarding how their symptoms might fluctuate over time (Bateman et al., 2023). The responsiveness analysis suggests that this would be an appropriate way to monitor treatment effect. The qualitative study also identified that the questionnaires were too time-consuming and needed to be shortened. We included two psychological measures: the TSK-11 measure of fear of movement and PSEQ questionnaire to capture pain self-efficacy, and found that the PSEQ was more highly correlated with treatment outcome. Similarly, we included both pain-free grip-strength and maximum grip-strength but the former was more highly correlated with treatment outcome. This is consistent with previous studies comparing the two methods (Macdermid and Silbernagel, 2015). Therefore, the TSK-11 and maximum grip-strength could be removed from a future main trial.

We piloted a method of grip-strength self-measurement using the Squegg device. Grip-strength data were provided in 77% of questionnaire returns, suggesting not all participants could/would use the device. Qualitative data suggested that some participants relied upon the assistance of family to use the device and one person was unable to connect it to their smartphone (Bateman et al., 2023). A previous UK study that used an analogue spring balance for similar self-measurement, reported 73% data return suggesting that other factors were involved, rather than the choice of device.

Adherence to exercise remains a challenge in physiotherapy trials and is difficult to measure (Mallett et al., 2020). The daily exercise diary that we piloted was only returned by a third of participants, so failed to provide meaningful data. The EARS questionnaire provided a complete dataset from questionnaires returned, so would be the preferred method of assessment of exercise adherence in a main trial.

Although the focus of this pilot and feasibility trial was not on between-group differences (and we did not conduct statistical tests to compare outcomes), the descriptive analysis of the data showed improvements in both groups in health outcomes over 6 months. In some outcomes, the trend was towards greater improvement in the usual care group than the intervention group for disability and perceived overall treatment effect, which was not expected. This may be explained by the usual care provided by the research-active sites involved in the trial being of higher quality than that provided by non-research-active centres more generally. Whilst a fully-powered future RCT would help ascertain whether the intervention is more clinically- or cost-effective than usual care, a future trial using the same intervention approach is unlikely to be desirable given the results of this pilot. A recent systematic review and meta-analysis of LET trials including placebo or wait-and-see controls suggested that following enrolment in a clinical trial, patients experience improvements over time without any active treatment, regardless of symptom duration prior to enrolment (Ikonen et al., 2022). It is important to note that the four included studies that were described as wait-and-see controls still included clinical assessment, a diagnosis of LET and provision of reassurance and advice. This may explain why improvements were seen in patients with longstanding symptoms after enrolment in research. Our qualitative interviews highlighted that patients were reluctant to follow self-help advice until they had received a diagnosis from a healthcare professional, reinforcing this hypothesis (Bateman et al., 2023). Careful consideration is therefore needed in terms of next research steps. Future randomised trials might instead want to consider comparing whether a single appointment to assess the patient, confirm diagnosis, reassure and provide self-help advice is non-inferior to usual physiotherapy interventions. The OPTimisE intervention could be adapted to form the self-directed advice and education component for such a trial.

The strengths of this study are that we were able to pilot different methods of participant identification, data collection and outcomes questionnaires, with clear quantitative and qualitative findings that allow us to refine the method of a future main trial. We included patient and public experience in our intervention and trial design. The design was limited by the lack of translation services, potentially resulting in fewer underserved communities being represented, but this could be addressed in the main trial design.

## 6 Conclusion

It is methodologically feasible to conduct a fully powered RCT to compare the clinical and cost-effectiveness of the OPTimisE treatment protocol against usual physiotherapy treatment. However, both intervention and usual care groups showed similar improvements over time, questioning the importance of a future comparative main trial. Future research might now consider comparing whether diagnosis, reassurance and comprehensive self-help advice is non-inferior to usual physiotherapy care in terms of cost-effectiveness. Any future trial would need to be adapted to simplify patient identification, shorten outcome questionnaires and include incentivisation, use online data collection by default, add monthly SMS text message outcomes, include minimum data collection by telephone, and incorporate language translation.

## Ethics approval and consent to participate

Approvals were received from the Yorkshire & The Humber - Sheffield Research Ethics Committee (reference 21-YH-0121) and the UK Health Research Authority (reference 297637) on June 22nd^,^ 2021. All participants were asked to provide written informed consent.

## Trial registration

Registered with the ISRCTN database 19/7/2021. https://www.isrctn.com/ISRCTN64444585.

## Funding

Marcus Bateman is funded by a 10.13039/501100000272National Institute for Health and Care Research (NIHR) and 10.13039/100011698Chartered Society of Physiotherapy Charitable Trust Doctoral Fellowship (reference NIHR300704).

## Patient and public involvement

Patient volunteers were involved with the design of the OPTimisE intervention, selection of orthosis from a range of available products, generation of trial website frequently asked questions and review of trial resources. KC is a member of both the OPTimisE Patient and Public Involvement Group and Trial Management Group, contributing to the trial design, trial website, trial management, interpretation of the data and writing of this report.

## Authors’ contributions

MB, CL, NF, BS, BV and DD were involved in the preliminary design and funding application. MB, CL, NF, BS, JCH, BV, JB, KC, AS and DD designed the trial protocol. All authors contributed to the conduct and management of the trial. MB, BS, CL, KC and JCH conducted the qualitative data analysis. MB and JB conducted the quantitative analysis. All authors contributed to this manuscript.

## Declaration of competing interest

The authors have no conflicts of interest to declare.

This paper presents independent research funded by the 10.13039/501100000272National Institute for Health and Care Research (NIHR) and 10.13039/100011698Chartered Society of Physiotherapy Charitable Trust. The views expressed are those of the author(s) and not necessarily those of Chartered Society of Physiotherapy Charitable Trust, the NHS, the NIHR or the Department of Health and Social Care.

## Footnotes

1 *PRTEE = Patient-Rated Tennis Elbow Evaluation, ranging from 0 (normal) to 100 (very high pain and disability). It has a minimal clinically important difference (MCID) of 11 points*.

2 *NRS = Numerical Rating Scale (0-10)*.

3 *GPE-11 = Global Perceived Effect 11-point scale, ranging from -5 (very much worse) to +5 (completely better)*.

4 *TSK-11 = Tampa Scale of Kinesiophobia (11 question version), ranging from 11 (no kinesiophobia) to 44 (very high kinesiophobia)*.

5 *PSEQ = Patient Self-Efficacy Questionnaire, ranging from 0 (very low self-efficacy) to 60 (high self-efficacy)*.

6 *EQ5D5L = EuroQol questionnaire (5 dimension, 5 level version)*.

7 *EARS = Exercise Adherence Rating Scale, ranging from 0 (very low exercise adherence) to 24 (very high)*.
