# Supplementary material for: Optimising physiotherapy for people with lateral elbow tendinopathy – Results of a mixed-methods pilot and feasibility randomised controlled trial (OPTimisE)
Source: Musculoskelet Sci Pract. 2024 Feb;69:None. doi: 10.1016/j.msksp.2023.102905 (PMC10843168; doi:10.1016/j.msksp.2023.102905)
Supplement: Multimedia component 1 [file mmc1.pdf]

OPTimisE

Optimising Physiotherapy for Tennis Elbow

Intervention Handbook

The OPTimisE intervention was developed using a combination of the best available research evidence and stakeholder consensus. It comprises of three treatment categories:

1. Advice and education
2. Exercise therapy
3. Orthotics

This handbook provides detailed instructions regarding how these treatments should be delivered. If you have any questions related to these instructions, please contact [redacted].

Treatment Process Map:

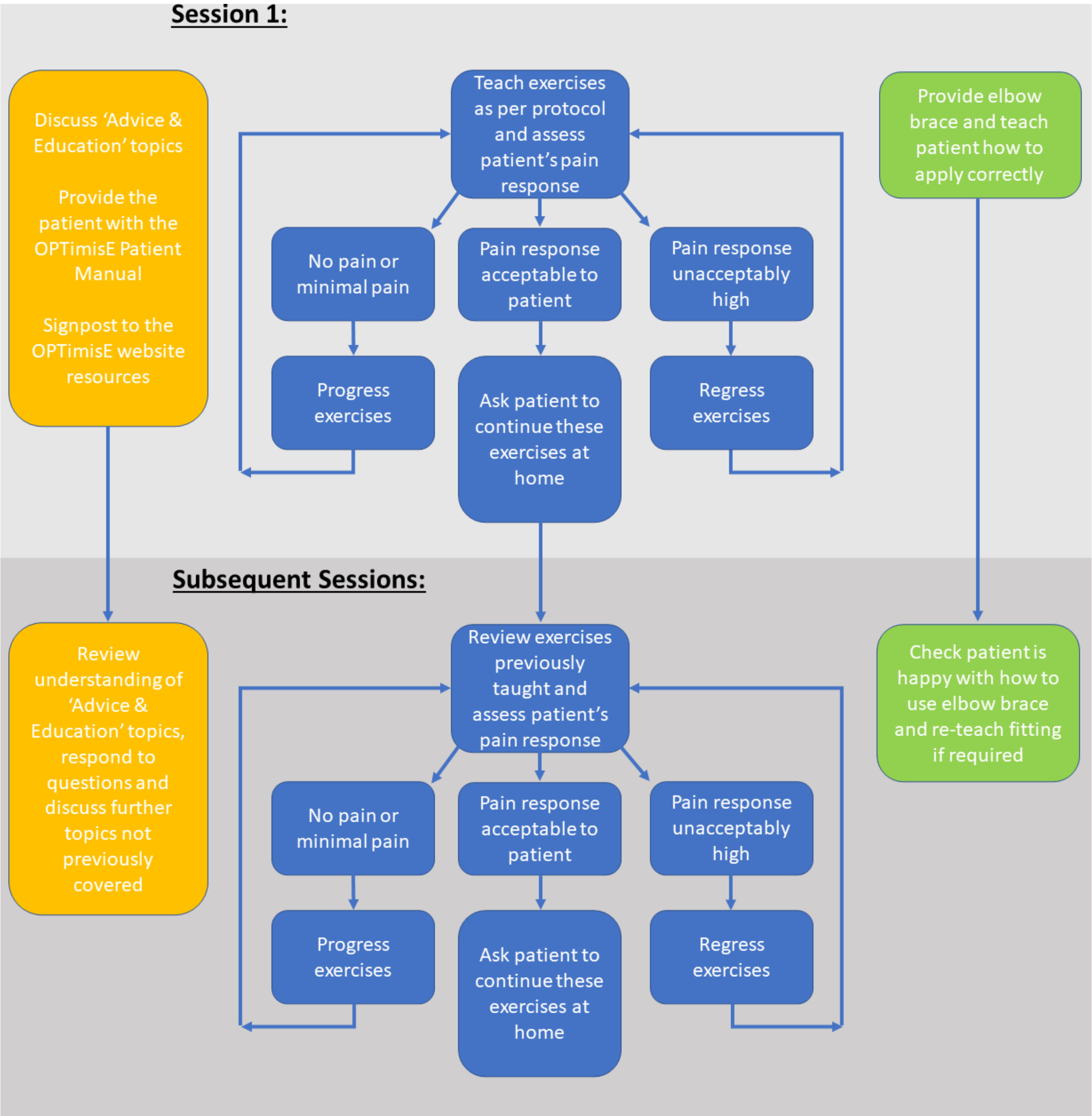

Advice and education

Discuss the following topics, if relevant to the patient, in order of priority, across treatment sessions. Provide patients with the 'OPTimisE Patient Manual' and encourage the patient to also visit the online resources on the OPTimisE website (www.optimise-trial.uk) for further information. Topics and patient understanding should be re-visited at subsequent sessions.

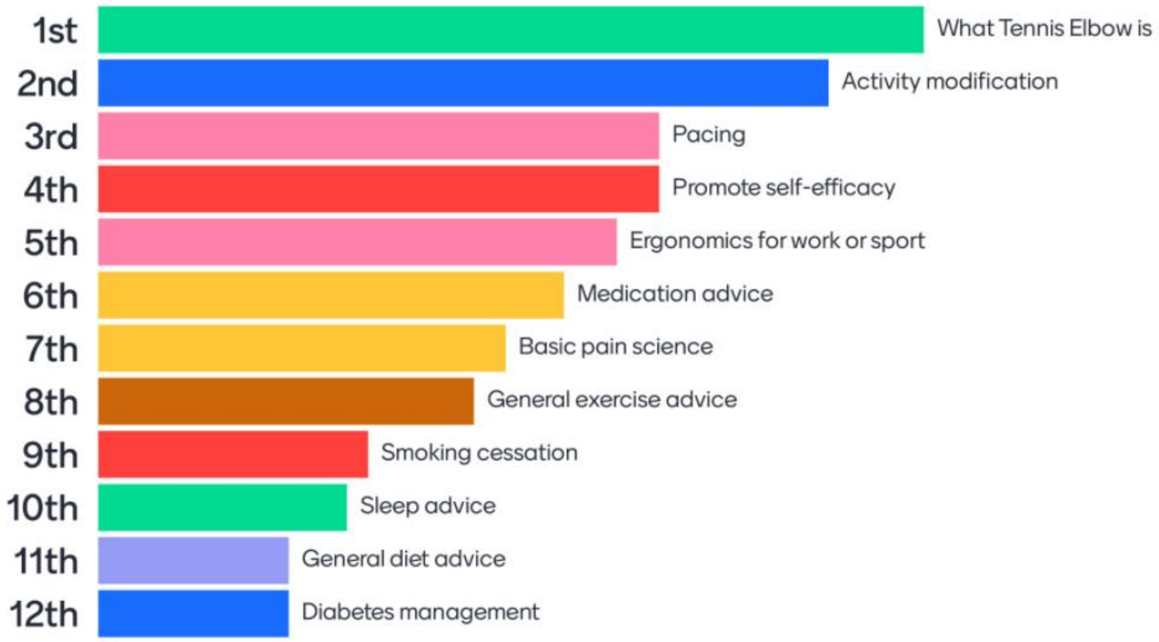

The following table advises on content for each topic:

Topic	Guidance
What tennis elbow is	Describe the condition as being 'pain from the tendons that move your wrist and help you grip'. Avoid using words that imply structural failure such as 'degeneration', 'damage' or 'disease'. Discuss common causes such as an episode of relative overuse, persistent heavy lifting or repetitive work involving the wrist & hands. Discuss age-related changes to metabolism that may be involved, as it most commonly occurs in middle age.
Activity modification	Discuss how painful tendons become very sensitive to the load that they are subjected to. Discourage trying to maintain normal activities despite high pain levels. Encourage modification of activities to reduce pain whilst still allowing function (e.g. palm-up lifting, use of the contra-lateral limb, use of lifting aids). Discuss the concept of relative rest but not complete rest. Encourage conversations with employers to adjust working routine if work is a significant contributory factor.
Pacing	Discuss the concept of pacing i.e. dividing larger tasks into manageable smaller stages interspersed with rest or other activities.

Promote self-efficacy	Self-efficacy is the patient's level of belief that they can overcome a problem. Discuss the importance of the patient understanding their condition, identify areas of their lifestyle that are affected and encourage them to think of solutions or ways of managing those situations. Reassure that those patients who have higher self-efficacy and take responsibility for managing their condition usually do better than those who rely on others.
Ergonomics for work or sport	Identify common work-related tasks that involve using the wrist in deviated positions, involve highly repetitive wrist movements or involve forceful gripping/wrist movements. Work with the patient to identify modifications or solutions.<br>For sports involving racquets, bats or sticks consider trying a larger grip or reducing tension in racquet strings. Try to grip less firmly and consider altering technique to reduce ulnar deviation. Consider a session with a qualified coach to assess/improve technique.
Medication advice	If pain is interfering with the patient's quality of life, advise regarding the use of analgesia based upon the World Health Organisation's analgesic ladder. Paracetamol +/- NSAIDs should be sufficient. Topical NSAID gels have fewer side-effects than oral NSAIDs. Direct the patient to see their pharmacist regarding NSAIDs due to potential contra-indications for use (e.g. asthma, gastric ulcer, anti-coagulation).
Basic pain science	Explain the concepts of acute and chronic pain. Relate chronic pain to systemic factors that influence the nervous system, such as fear, stress, lack of sleep. Reassure that, in chronic pain situations, pain does not equal tissue damage. Encourage patients to work around their pain symptoms rather than avoiding painful activities totally.
General exercise advice	People who take regular exercise 2-3 times a week are 20% less likely to develop Tennis Elbow than those who don't. Encourage regular exercise to help promote healing, improve general health and mental-wellbeing. Further information on NHS Get Active website.
Smoking cessation (if applicable)	Research has shown that smokers are up to 3.6 times more likely to develop tennis elbow than non-smokers. Stopping smoking is likely to improve the chances of recovery. Please direct patients to local smoking cessation services or NHS Quit Smoking website.
Sleep advice	Poor-quality or lack of sleep can cause increased sensitivity to pain and lead to patients experiencing chronic pain. Discuss the patient's sleep patterns and advise regarding avoidance of caffeine, nicotine, opioid medication and alcohol before bed. Promote regular exercise. Encourage regular bedtime and wake-up times for routine. Avoid daytime naps. If sleep is disturbed by background noise consider earplugs or 'white noise' mobile phone apps.
General diet advice	Promote a healthy balanced diet rich in fruit, vegetables and fibre. High energy diets have been associated with inflammation and chronic pain. Obesity is an independent predictor of chronic pain. Obese patients should be advised to seek help from their GP or dietician. Further information on NHS Lose Weight website.

Diabetes management (if applicable)	People with diabetes are up to 2.1 times more likely to develop Tennis Elbow than those without. Poorly controlled diabetes is associated with slower tissue healing. Discuss the patient's diabetes control and advise to consult their diabetes practitioner if concerns are highlighted.

Exercise Therapy

A progressive exercise regime should be taught to all patients. No more than four exercises should be provided at any one time and the total exercise time should not exceed 15 minutes. Patients should aim to perform one session of exercise per day and continue over a minimum period of 12 weeks. Exercises should provoke pain to a level that is acceptable to the patient, both at the time of performing the exercises and during the subsequent 24 hours. The level of exercise should be adapted to the severity of the patient's symptoms but patients should aim to tolerate high level exercises before discharge.

Low level exercises:

- Forearm stretches – elbow extended, forearm pronated, wrist flexed and ulnar-deviated. Stretch into moderate discomfort as acceptable to the patient. 30s hold x 3, before and after loading exercises.

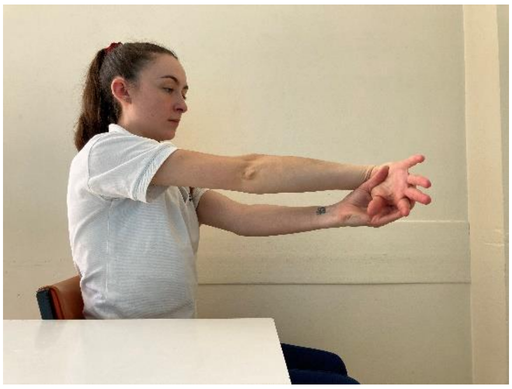

- Isometric loading – sit with elbow flexed to 90°, forearm supported on a table and pronated. A maximal isometric contraction is performed (based upon acceptable level of pain) in full wrist extension, using the opposite hand to apply the resistance. Hold for 60 seconds and repeat 5 times.

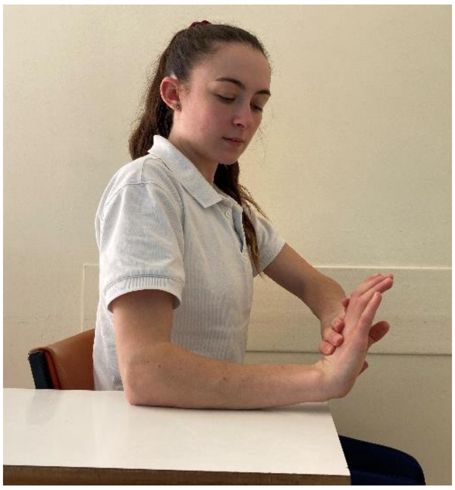

Medium level exercises:

- Combined concentric and eccentric loading – the elbow should be fully extended with the forearm supported and pronated. Starting from a fully flexed wrist position a concentric exercise is performed to the full range of wrist extension. This movement should be done slowly over at least six seconds. A slow eccentric exercise is then performed over six seconds to return to the starting position. Three sets should be performed 10-15 times with a minute's rest in between sets. The amount of load should be varied due to the individual patient's pain response. This may vary day-to-day and patients should be taught to modify the load themselves accordingly. Load can be applied using weights (including household objects) or elasticated exercise bands. *Note: if the exercise is too painful, try repeating with the elbow flexed to 90° rather than fully extended. Also, be aware that loading with a large size object may be more painful than with a smaller object of the same weight.*

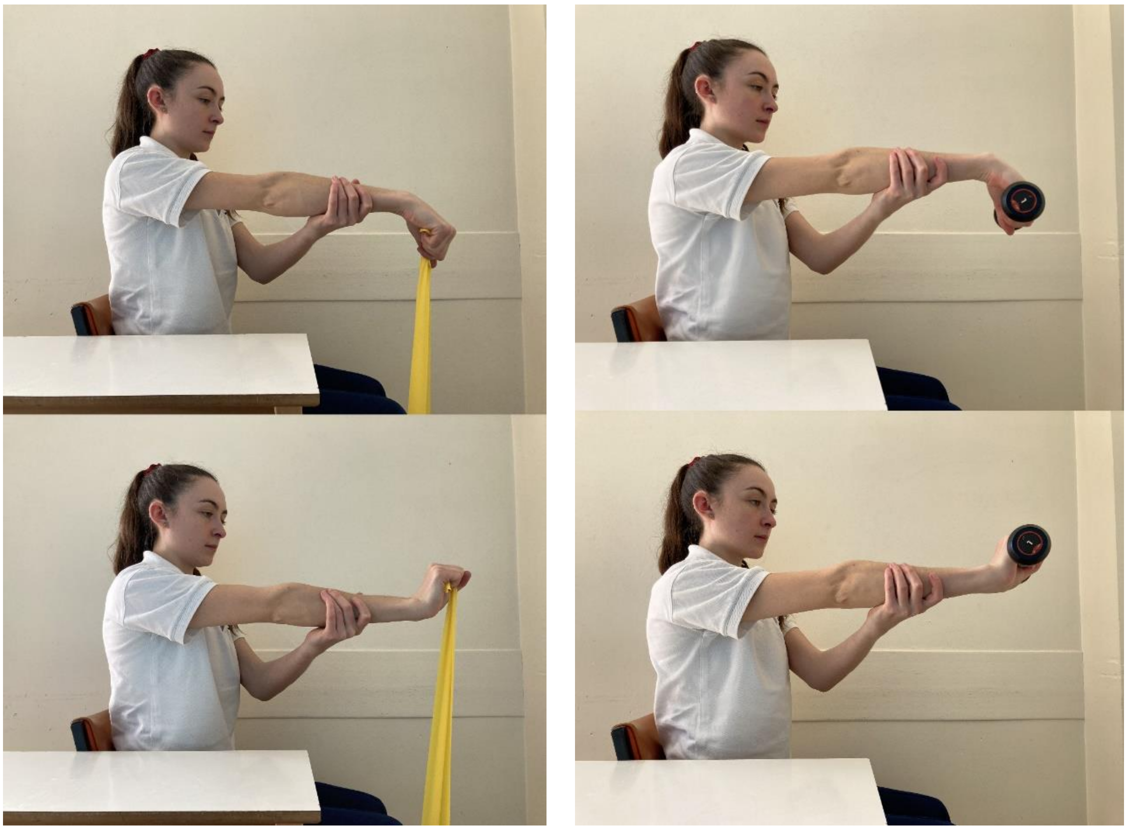

High level exercises:

- Functional higher load exercises tailored to the patient’s activity demands - For example, plyometric racquet sport simulation with resistance or high load simulation of work activities.
- Higher load general upper body exercise - For example: incline push-ups, kneeling push-ups, full push-ups, shoulder press, bent-over rows.

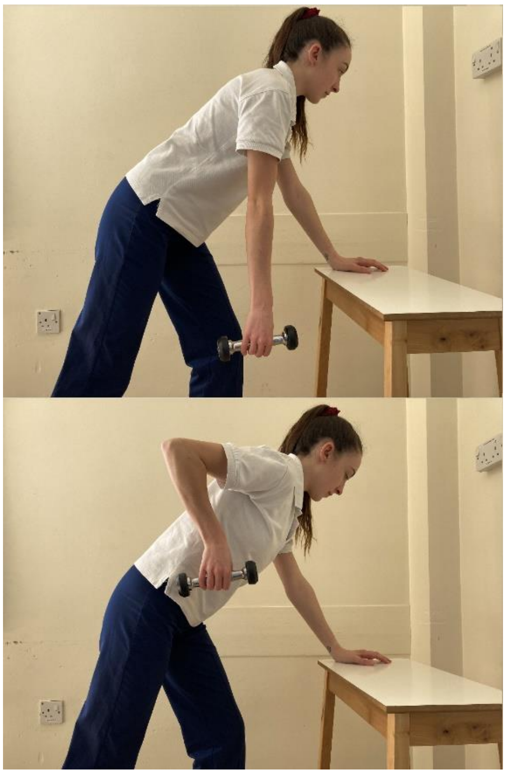

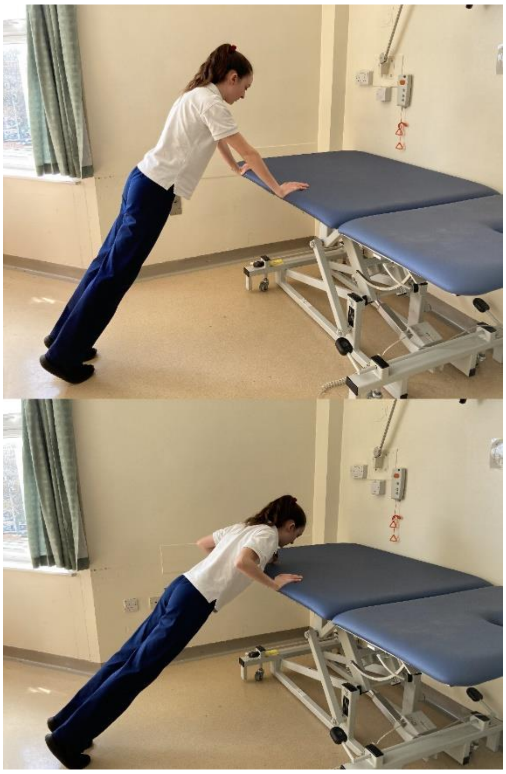

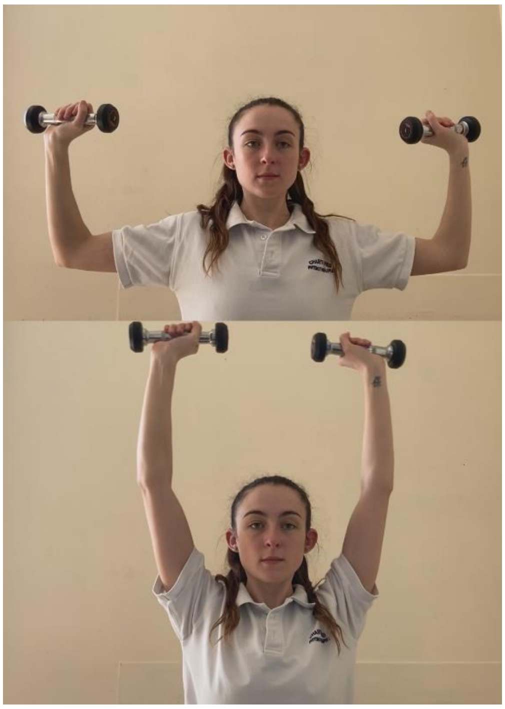

Orthotics

Patients should be provided with a counterforce brace to use if they wish. They should be instructed in the correct fitting of the brace and to only wear it during activities that regularly provoke significant pain. They should be discouraged from wearing it all of the time or during minimally painful activities to reduce reliance on the device. A video of how to fit the brace is available on the www.optimise-trial.uk website.
